# The Effect of the Parameters of T-RTM on the Properties of Polyamide 6 Prepared by in Situ Polymerization

**DOI:** 10.3390/ma13010004

**Published:** 2019-12-18

**Authors:** Orsolya Viktória Semperger, András Suplicz

**Affiliations:** 1 Department of Polymer Engineering, Faculty of Mechanical Engineering, Budapest University of Technology and Economics, Műegyetem rkp. 3, H-1111 Budapest, Hungary; orsolya.semperger@hd-composite.com; 2 HD Composite Zrt., Újszász utca 45, 1165 Budapest, Hungary

**Keywords:** in situ polymerization, T-RTM technology, polyamide 6, caprolactam, anionic ring-opening polymerization, crystallinity, monomer concentration, mechanical characterization, surface hardness

## Abstract

With the rapid development of the automotive industry, there is also a significant need to improve the raw materials used. Therefore, the demand is increasing for polymer composites with a focus on mass reduction and recyclability. Thermoplastic polymers are preferred because of their recyclability. As the automotive industry requires mass production, they require a thermoplastic raw material that can impregnate the reinforcement in a short cycle time. The most suitable monomer for this purpose is caprolactam. It can be most efficiently processed with T-RTM (thermoplastic resin transfer molding) technology, during which polyamide 6 is produced from the low-viscosity monomer by anionic ring-opening (in situ) polymerization in a tempered mold with a sufficiently short cycle time. Manufacturing parameters, such as polymerization time and mold temperature, highly influence the morphological and mechanical properties of the product. In this paper, the properties of polyamide 6 produced by T-RTM are analyzed as a function of the production parameters. We determine the crystallinity and the residual monomer content of the samples and their effect on mechanical properties.

## 1. Introduction

Nowadays, the automotive industry is developing very rapidly. Almost 100 million cars roll off the production lines every year worldwide. In order to keep up with this rapid development, we need to develop new materials. Interest in polymer composites has increased recently. There are number of studies where the goal is to develop materials and their composites which have such low density and good mechanical properties (such as high strength or stiffness), that they can even be used as load-bearing parts. The automotive industry uses a large proportion of these composites [1,2,3].

Therefore, mass production and a sufficiently fast production cycle time are essential for automation. The energy use and recyclability of the manufactured products also need to be considered, which justifies the use of a thermoplastic polymer matrix for composites. The spread of thermoplastic composites is inhibited by the high viscosity of the matrix (10–100 Pas) during the process, therefore, it cannot properly impregnate the continuous reinforcing material. A solution to this problem can be the use of caprolactam, which has low melt viscosity (3–5 mPas) and can be used to produce polyamide 6 by in situ polymerization. In the process, caprolactam is combined with the initiator and activator system. Polyamides are technically significant polymers. Their popularity is due to their favorable technical properties, such as excellent impact resistance, high abrasion resistance, and good strength properties. Research has, so far, mainly focused on developing and accelerating the polymerization process. Recently, impregnation tests have become widespread for various reinforcing fabrics: glass, carbon, etc. [4]. One of the most suitable technologies for the processing of caprolactam is the further developed version of combined RTM (resin transfer molding) technology. A highly efficient automated processing technology for the process is T-RTM (thermoplastic resin transfer molding). T-RTM is a thermoplastic injection process in which ring-opening in situ polymerization is carried out in a tempered mold at low pressure, with a sufficiently short cycle time. During the process, the preformed reinforcing material is placed into a closed mold and then the capolactam monomer with the initiator system is injected into the mold. The challenge of this manufacturing technology is to achieve a sufficiently short cycle time for automation [5]. Factors influencing the mechanical properties of the product include tool temperature and polymerization time [4]. The temperature of polymerization significantly influences the degree of crystallinity, conversion, molecular weight, and, indirectly, the thermomechanical properties of the product. The anionic polymerization of lactams can be executed in two temperature ranges: below or above the melting temperature (T_m_) of the polylactams. The melting temperature (T_m_) of PA6 is between 210 °C and 225 °C. Polymerization below Tm is preferred because it results in a high conversion rate (96–99%) and high crystallinity (40–50%). One of the most important advantages of polymerization under Tm is its relatively short cycle time and energy efficiency. Polymerization above Tm can result in relatively high residual monomer concentrations (up to 8–10 m%) [4,6,7].

Vicard et al. [8] found by using DSC that the polymerization of the monomer occurs simultaneously with its crystallization during the growth of the polymer chain. At high temperatures, polymerization precedes crystallization as there is not much cooling, therefore the crystalline fraction is reduced significantly. This finding was also confirmed by Bersee et al. [9]. They found that at too low a temperature, crystallization is too fast and there is not enough time for all the monomers to react.

Ben et al. [10] investigated the effect of the manufacturing settings of VARTM (vacuum assisted resin transfer molding) on the mechanical properties of in situ polymerized PA6 composite sheets. They used DSC to determine crystallinity and found that the crystalline fraction of in situ polymerized PA6 sheets decreases significantly with increasing mold temperature. At 140 °C, the crystalline fraction is about 40%, the proportion of unreacted monomer is 0–2 m%, while at 200 °C the crystalline fraction is reduced to 25% and monomer weight is about 5 m% [10]. The high proportion of crystals in in situ polymerization is because crystallization occurs simultaneously with polymerization. There are no molecules with a high molecular weight, therefore, the crystals can grow much more freely than in the case of re-melting, when the polymer molecules with a large molecular weight prevent the growth of a crystalline structure. The researchers found the specimens produced at higher temperatures weaker because of their high residual monomer content. The ideal temperature was found to be 160 °C [10].

Rijswijk et al. [11] found that polymerization happens below the melting temperature of PA6, therefore polymerization and crystallization occur at the same time, with a mold temperature of 140–170 °C. The highest achievable crystalline fraction during polymerization at 145–150 °C is about 50% [12].

Chun et al. discovered that with the increase of residence time, the degree of crystallization first increases, then becomes constant [13]. In studies so far, polyamide 6 samples have been prepared from caprolactam in a laboratory.

The aim of our study is to analyze how polyamide 6 can be produced with the T-RTM technology and to investigate the effect of the main technological parameters on properties of the product. We varied the temperature of the mold (but kept it below T_m_) and the residence time. We produced specimens with three different residence times.

## 2. Materials and Methods

### 2.1. Materials

The specimens were prepared from a system of ε-caprolactam (94%), C10 (sodium caprolactam) (3%) and C20P (hexamethylene-1,6-dicarbamoyl caprolactam) (3%), which is suitable for the production of anionic ring-opening polymerization of PA 6 with a short cycle time and below the melting point (130–170 °C). The melting point of AP-Nylon ε-caprolactam (L. Brüggemann GmbH and Co. KG, Heilbronn, Germany) is 69 °C, its density is 0.6–0.7 g/cm^3^ and its viscosity is similar to that of water (3–5 mPas). It was stored under vacuum because it has good moisture absorbency. Sodium caprolactam (Brüggolen C10, L. Brüggemann GmbH and Co. KG, Germany) was used as the initiator. Its density is 0.45–0.55 g/cm^3^ and its melting point is 62.2 °C. It is sensitive to moisture and is easily deactivated in the presence of water. Therefore, it was stored under vacuum too. Hexamethylene-1,6-dicarbamoyl caprolactam (Brüggolen C20P, L. Brüggemann GmbH and Co. KG, Heilbronn, Germany) was used as the activator. Its density is 0.85 g/cm^3^ and its melting point is 70 °C.

### 2.2. Sample Preparation

**Figure 1:**
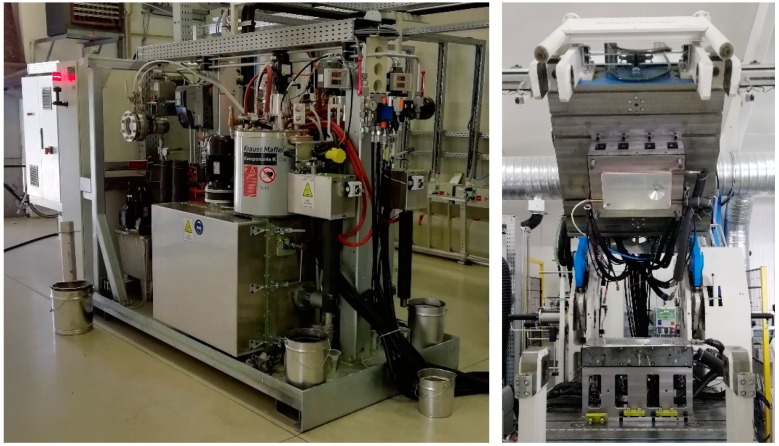
TRTM unit for in situ polymerization (dosing unit and hydraulic press).

The plate-shaped specimens (290 × 510 × 5.5 mm) were prepared by in situ polymerization with the use of the T-RTM (thermoplastic resin transfer molding) technology. We used KraussMaffei technology to produce the specimens. The equipment consists of Dosing Unit (DU) and a hydraulic press (Figure 1) The DU was heated up to 110 °C to melt the raw materials and provide the inert nitrogen atmosphere. A DU has two tanks for melting CL + C10 and CL + C20P separately.

After the components have been melted, they flow through separate heated pipes to the forming mold installed on the press. The mold was filled through a mixing head heated to 110 °C. The components come into contact with each other after the mixing head in the cavity, to prevent premature polymerization. The appropriate mixing of the components is provided by the design of the mixing head and the flow rate. The hermetically-sealed tool is under vacuum to ensure the best possible filling. After the tool was filled and polymerization time passed, the sample was removed.

### 2.3. Design of Experiments

We performed experiments on the crystalline ratio and residual monomer content of the specimens and investigated their effect on mechanical properties. Our literature research revealed that the most suitable temperature range is 140–190 °C and a polymerization time of 3–5 min. The recommended mixing ratio is 94% caprolactam, 3% initiator, and 3% activator—this ensures the highest conversion polymerization [10,11,13,14,15]. Five samples each were produced and examined with these test parameters.

Table 1 shows the manufacturing technology parameters set on the T-RTM machine. Two independent variables were used in the experiments: mold temperature and residence time. Mold temperature is the temperature of the tool during production. Residence time is the time from the injection of the material into the mold until the removal of the finished sample. Sufficient time must be allowed for polymerization during production, otherwise the process will not achieve a sufficiently high conversion, and the properties of the material will be inferior. During production these parameters determine the morphological structure of the material produced (molecular weight, crystalline structure and proportion, crystal size, etc.) and, therefore, its mechanical and thermal properties. The samples we studied do not contain any additives other than the initiator and activator, so the results obtained are the properties of the unfilled material.

### 2.4. Characterization of the Samples

The residual monomer content of the samples produced with different technological parameters was determined by TGA tests. The tests were performed on a TGA Q500 instrument (TA Instruments, New Castle, USA) at a temperature between 50 °C and 350 °C with a heating rate of 10 °C/min. The crystalline fraction of the samples was determined with a TA DSC Q2000 device (TA Instruments, New Castle, USA) (1). We performed the test in a heat-cool-heat cycle between 25 °C and 250 °C with a heating and cooling rate of 10 °C/min. The first heating provided information about the conditions of production, and the second heating yielded properties of the material. The crystalline fraction of the samples was determined as follows:

(1) $$X=\frac{\Delta H_{m}-\Delta H_{cc}}{\Delta H_{f}\cdot (1-\varphi )},$$

where Δ*H_m_* is the enthalpy of melting, Δ*H_cc_* is the enthalpy of cold crystallization, Δ*H_f_* is the melting enthalpy of a theoretically fully crystalline polymer and ϕ is the mass fraction of the filler. We used Δ*H_f_* = 188 J/g [16] in the case of PA 6.

The Shore-D surface hardness was determined with a ZWICK H04.3150.000 device (Zwick GmbH and Co. KG, Germany). The three point bending test was performed with a Zwick Z020 computer-controlled universal loading machine (Zwick GmbH and Co. KG, Germany) with a bending speed of 10 mm/min, following the recommendations of ISO 178: 2011. The specimens were not damaged during the test; they were loaded to their bending limit. The size of the samples was 130 × 15 × 5.5 mm and the support distance was 104 mm. We tested dynamic mechanical properties with a Charpy impact test, using a Resil Impactor Junior (Ceast, Italy) with a 15 J hammer. The recommendations of ISO 179 were taken into account in the measurements and evaluations. The size of the samples was 80 × 10 × 5.5 mm with a Type B cut. The support distance was 62 mm.

The ductility index was determined from Equation (2). The ductility index is the quotient of the E_Fmax_ and E_total_ impact energies. It characterizes the type of failure. Its value is between 0 and 1.

(2) $$DI=\frac{E_{F\max}}{E_{total}},$$

DI is the ductility index (-), E_Fmax_ is the impact work absorbed until maximum force is reached (J), and E_total_ is the impact work absorbed until the first zero transition is reached (J).

## 3. Results

### 3.1. Influence of Technological Parameters on Crystallinity

**Figure 2:**
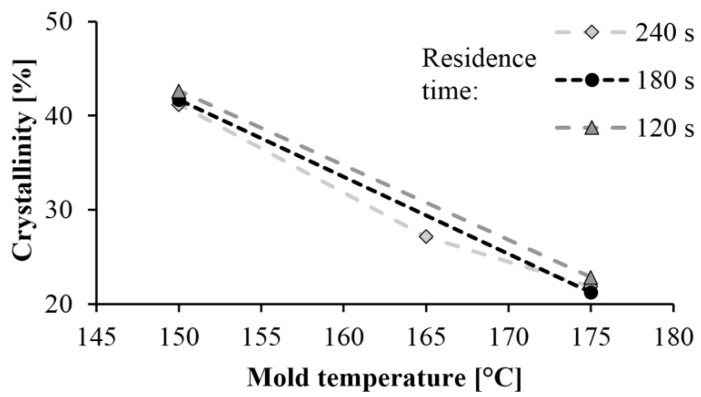
Crystallinity of samples based on first heating.

**Figure 3:**
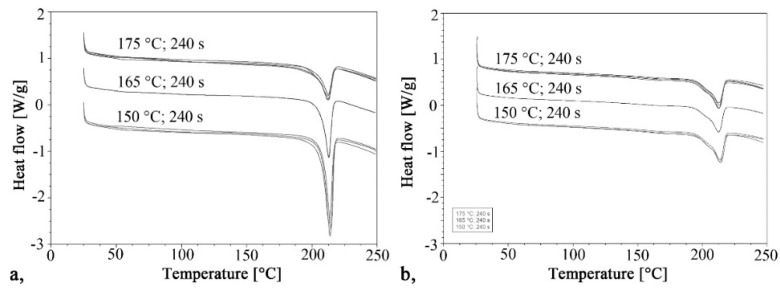
Heating curves for the DSC test: (**a**) First heating, (**b**) Second heating.

The curves of the first and second heating obtained by DSC are shown in Figure 2 and Figure 3 shows the crystalline fraction of the first heating. The first heating curves show that crystallinity increases significantly as mold temperature decreases. This value is approximately 40–43% in samples made with a 150 °C mold. It decreased almost linearly with the increase of temperature in the tested range, and it was 20% when mold temperature was 175 °C. This is the same as the crystalline fraction of PA 6 crystallizing from a melt state. One of the possible reasons is that it is more difficult for hydrogen bridge bonds to form because of the greater thermal motion of the particles at higher temperatures. Furthermore, as temperature increases, the side reactions during polymerization also increase, which in turn increases the number of irregular polymer chains. These irregularities also hinder the crystallization process.

The curves of the second heating are very similar to each other, as each sample crystallized the same way, from a melt state. The crystalline fractions varied between 18.4% and 21.5%. The maximums of the crystal melting peaks were also the same; their values were between 212 °C and 214.4 °C in both the first and second heating.

Residence time did not significantly influence the crystalline fraction. One of the possible reasons is that the specimens cooled in the open air after the opening of the mold, and it caused post-polymerization. The samples were still at a high temperature they were removed from the mold, due to the poor thermal conductivity of the polymers and the thickness of the samples, which was 5.5 mm.

The crystalline fraction influences many properties of the material. A higher crystalline fraction means increased hardness and modulus, improved chemical resistance, and increased water resistance and toughness [17]. We examined the effect of the crystalline fraction on the properties of PA6 produced by anionic ring-opening polymerization.

### 3.2. Monomer Concentration of the Samples

Residual monomer means the unreacted monomer during polymerization, which reduces the final conversion rate. If this value is greater than 4–5%, it is necessary to remove it from the polymer because CL behaves as a plasticizer inside the material and some of it condenses on the surface of the product where it prevents coating it.

TGA was used to determine the residual monomer fraction. We tested samples conditioned at room temperature (23 °C) and a humidity of 38%. Increasing temperature causes weight decrease which is the result of the degradation or leaving of different materials. The process occurs in different temperature ranges for different materials. According to Yang et al., three different temperature ranges can be separated. The first (80–100 °C) is where water evaporates from the sample. The next is between 100 °C and 250 °C, where the various solvents and small molecules (such as monomers, oligomers) leave. Finally, the polymer degrades—the chains break up—above the melting temperature, and as temperature approaches decomposition temperature, the material starts to decompose [18].

**Figure 4:**
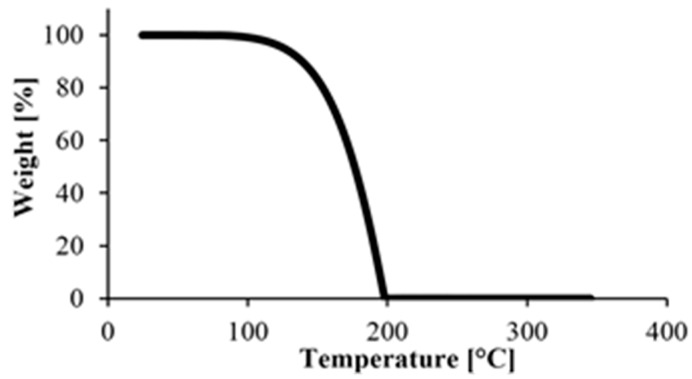
TGA measurement of the starting monomer.

First, we performed a TGA test on caprolactam, with the results in Figure 4. The material degraded completely below 200 °C, which is below the melting point of PA. This was good because TGA tests can only be used to determine the amount of monomer remaining in the polymer if there is no monomer left in the specimens when degradation begins.

**Figure 5:**
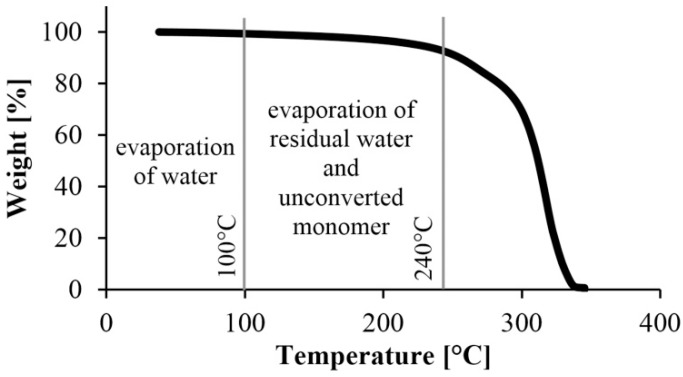
A typical TGA curve to determine the remaining monomer concentration.

**Figure 6:**
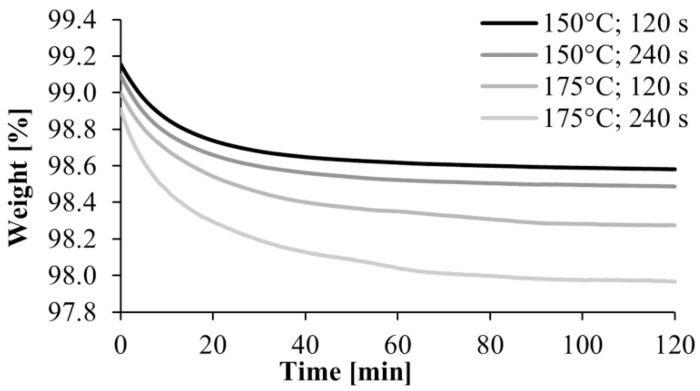
TGA curves of the drying of polyamide 6 samples.

After the preliminary experiment, we analyzed under nitrogen gas the polyamide samples produced according to the experimental design. A typical TGA curve is shown in Figure 5. It shows a break at about 250 °C, which is where the material begins to degrade, and another major break at about 300 °C, where the degradation of the material is significantly accelerated. Based on this result, we used the weight percentage of the samples at 240 °C in the evaluation. It was corrected with the water content obtained from the preliminary experiments, where the samples were kept at 80 °C for 2 h in the TGA apparatus (Figure 6). The water content of the monomer was subtracted from the weight of the polymer and the weight of the monomer was obtained.

**Figure 7:**
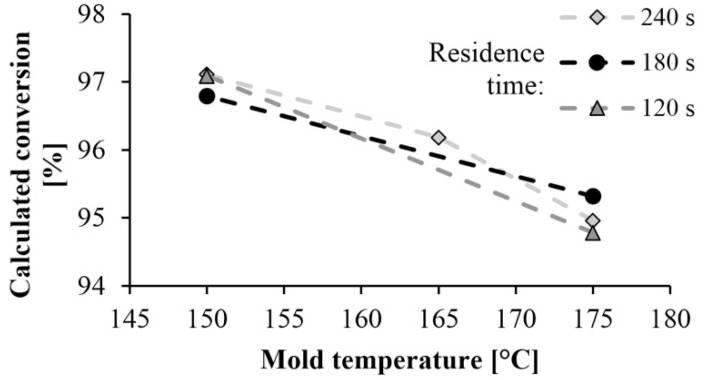
Dependence of monomer proportion on mold temperature and residence time.

Figure 7 shows the monomer content remaining in the polyamide as a function of mold temperature and residence time. The high conversion rate of 95–97% obtained at a mold temperature of 150 °C resulted in a low residual monomer content. It is very beneficial from the point of view of manufacturing technology. Monomer fraction decreases with the increase of mold temperature, which can occur for two reasons. The DSC test showed that lower mold temperature results in higher crystallinity, which increases the final conversion rate, as the crystalline phase can be considered 100% conversion. Furthermore, at lower temperatures the monomer-polymer thermodynamic equilibrium shifts toward polymer, which also increases conversion. Varying the residence time has no significant effect on the test results.

### 3.3. Mechanical Characterization

Using the cross-sections, we calculated the bending stress and the bending modulus from the force and displacement values. The test was continued until the boundary bending was reached, which is 10% of the support distance; in this case, it was 10.4 mm. Classical calculating methods can be considered valid up to this value. Since the specimens did not break during the bend test, we were only able to determine the limit bending stress. This value is proportional to the maximum load capacity. Six specimens with the same parameters were used for the tests.

**Figure 8:**
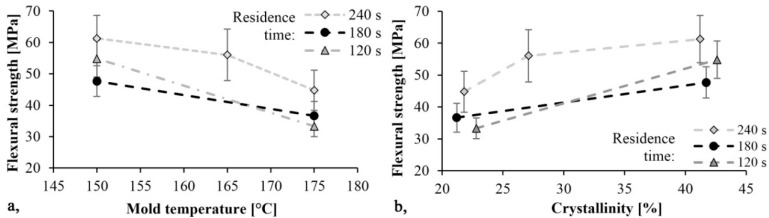
Bending stress as a function of mold temperature (**a**) and crystallinity (**b**).

**Figure 9:**
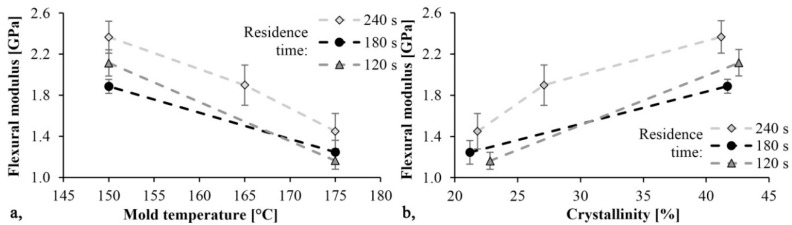
Young modulus as a function of mold temperature (**a**) and crystallinity (**b**).

The trends in Figure 8 and Figure 9 are the same. As mold temperature decreases, bending stress and Young modulus increase, which means that the material becomes stiffer. Based on previous studies this is probably due to the higher crystalline fraction and residual monomer content. The maximum limit bending stress was 61 MPa and it decreased by 30%, while the maximum Young’s modulus was 2.4 GPa and it decreased by 40% when mold temperature was increased from 150 °C to 175 °C. In both cases, the maximum values were reached with a mold temperature of 150 °C and a residence time of 240 s. In this experiment, the residence time had a significant effect. Increasing residence times resulted in increasing bending stress and Young’s modulus.

The Charpy impact bending tests were performed on notched specimens. The E_Fmax_ and E_total_ impact energy values were recorded. E_Fmax_ was recorded until F_max_ was reached and E_total_ represents the area under the first zero transition curve. α_C_ can be calculated from the A_0_ cross section—reduced by the notch—and the E_Fmax_.

**Figure 10:**
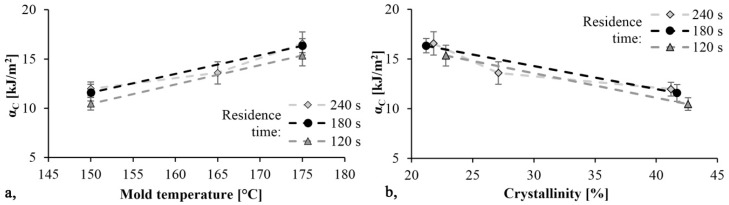
Impact strength as a function of mold temperature (**a**) and crystallinity (**b**).

The specific impact values are shown in Figure 10. As mold temperature increases α_C_ increases as well and the material becomes tougher, while the Young modulus decreases. Additionally, as the TGA test showed, increasing the temperature increases monomer content. The monomer has a softening effect on PA 6 and so it can increase toughness. The maximum specific impact strength was 16.6 kJ/m^2^ and it was achieved with the specimen produced with a mold temperature of 175 °C and a residence time of 240 s. Specific impact work decreased slightly as reaction time decreased.

**Figure 11:**
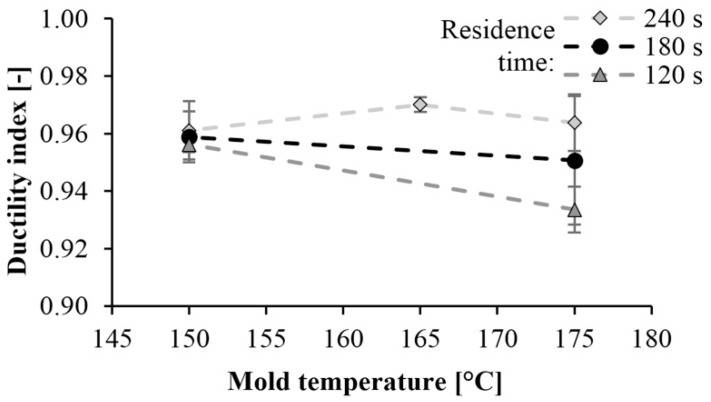
Dependence of the ductility index on mold temperature and reaction time.

Then we determined the ductility index. The ductility index of PA 6 is shown in Figure 11 as a function of mold temperature. This value is between 0.92 and 0.98. Values near 1 indicate brittle fracture. The tougher the material is, the less the ductility index is. When examining the fracture surfaces, we found a straight fracture in all cases. No permanent deformation was left in the material, which could have caused energy absorption. This also indicates a brittle fracture.

### 3.4. Surface Hardness

**Figure 12:**
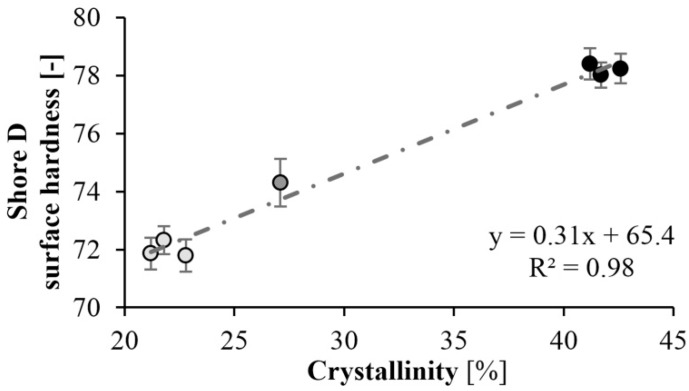
Hardness as a function of crystallinity.

**Figure 13:**
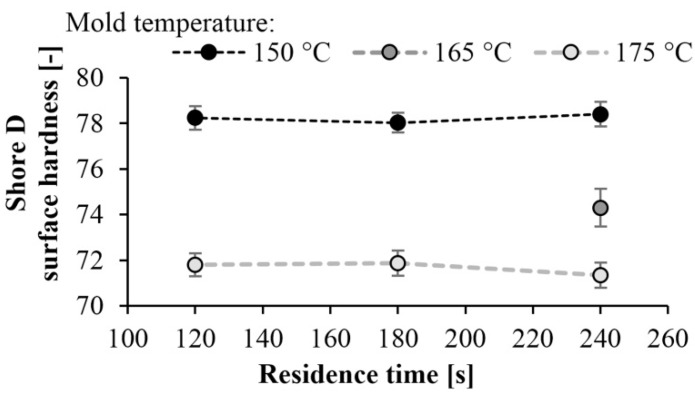
Hardness as a function of residence time at different temperatures.

Surface hardness tests (Shore-D) were also performed on the specimens. As expected, the hardness of the specimens increased as mold temperature decreased. A maximum of hardness of 78 Shore D was achieved at a mold temperature of 150 °C. There is a significant difference in hardness at different temperatures. At lower mold temperatures, the crystalline fraction is higher, which increases hardness (Figure 12). The results are consistent with the DSC test. At a mold temperature of 150 °C a crystalline fraction of 42–43% can be achieved, which results in a Shore D hardness of 78. Increasing the mold temperature to 175 °C causes an 8% reduction in hardness. Similarly to the DSC test, reaction time did not have a significant effect (Figure 13).

## 4. Conclusions

We prepared polyamide 6 samples from caprolactam by in situ polymerization, using the T-RTM technology. We studied the effects of various manufacturing parameters, such as mold temperature and residence time. We measured the crystalline proportion and residual monomer content of the polyamide 6. In addition, we studied the effect of crystallinity and residual monomer content of the specimen on the mechanical properties and surface hardness of the product.

First, we determined the residual monomer content of the samples. The result was that the monomer fraction decreases with increasing tool temperature. Increasing mold temperature significantly reduced the crystalline proportion of the product. The crystalline proportion was 40–43% at 150 °C and 20% at 175 °C. We performed further tests because crystallinity can influence many other properties of the material. The three-point bending tests showed that bending stress and flexural modulus increase with decreasing mold temperature. As the specimens did not break during the test, the limit bending stress was determined. The maximum limit bending stress was 61 MPa and it decreased by 30%, while the maximum flexural modulus was 2.4 GPa and it decreased by 40% when mold temperature was increased from 150 °C to 175 °C. We determined the dynamic mechanical properties of the samples with a Charpy impact test. When mold temperature increases, α_C_ also increases, and the material becomes tougher. The maximum specific impact work was 16.6 kJ/m^2^ when residence time was 240 s and mold temperature was 175 °C. Finally, we performed a Shore D surface hardness test. The hardness of the specimens increased as temperature decreased. This is consistent with the crystalline proportion of the sample. Maximum hardness was 78 Shore D at 150 °C (42–43% crystalline proportion). The hardness of the samples decreased by about 8% as mold temperature increased to 175 °C. Finally, we pointed out that residence time did not significantly influence the measurement results. We proved that the mold temperature, hence the polymerization temperature, had the greatest effect on the properties of the part. It can influence crystallinity, mechanical properties, and the conversion of the material. In contrast to the literature data, residence time had no significant effect during T-RTM, because polymerization continues after ejection, as the temperature of the part is high enough.

## Figures and Tables

**Table 1:** Manufacturing parameters.

Residence Time	Temperature of the Mold		
	150 °C	165 °C	175 °C
120 s	X	X	X
180 s	X		X
240 s	X		X
